# False Impressions

**Published:** 1888-10-06

**Authors:** Caroline Fothergill

**Affiliations:** Author of "An Enthusiast," "A Voice in the Wilderness," etc.


### False Impressions.

By Caroline Fothergill, Author of "An Enthusiast," "A Voice in the Wilderness," etc.

### Chapter I.—Mrs. Lennox's Garden Party.

Mrs. Lennox's garden party was at its height; she had sent out many invitations to it, and almost all had been accepted. That was only what she had expected; her garden parties were justly renowned, and she was a lady totally unaccustomed to social failure. All her guests were assembled except Miss Stanford and Mrs. Ledward, a proud and handsome young heiress, and the widowed schoolfellow who acted as her chaperon, though she was undeniably more lively and frivolous than was her charge. While she wondered what detained them, on turning her head she saw them coming towards her, Miss Stanford walking with the light, free step, which was suggestive of speed without hurry, and Mrs. Ledward looking like a bright, vivacious bird. Mrs. Lennox greeted them with effusion; it was the first time Miss Stanford had been at her house, and she was anxious to become intimate with her; she kept her, therefore, for some minutes in conversation, and only released her when someone else claimed her attention.

Summer had come late that year, and it stayed late. September—the month when this story opens—was warmer and more balmy than June had been, and tennis and garden parties were still the most popular form of entertainment. This particular afternoon was exquisite, the gardens looked gorgeous, the guests were many and gay. Beatrice Stanford, in a yellow brown dress which suited her perfectly, and a large wavy hat, which also became her perfectly, was amongst the gayest. She was in good spirits, gracious, and happy beyond her wont, and on leaving her hostess was soon the centre of the group which always formed around her wherever she went.

Mrs. Ledward, her companion, had her group too, of a totally different character. Whenever she and Beatrice went out together they were always separated at once, and often did not meet again until it was time to leave. On this occasion they never met once, a circumstance for which Beatrice afterwards felt very grateful.

The group, of which she was the centre, was constantly changing; new people were added to it, others strolled away. The subject of conversation changed too, and by-and-by fell upon an outbreak of smallpox in a neighbouring town, the origin of which could not be satisfactorily settled. Someone prophesied that Mayfield, a notoriously unhealthy town, would have its turn in the winter. Miss Stanford listened with composure and some amusement to the fears expressed. She was not naturally nervous, and had great faith in her own sound constitution and her ability to resist epidemics. She said you only needed to keep cool and lead your accustomed life. But this was not the opinion of a lady who sat near her, an anxious, rather hysterical looking woman, who, although her acquaintance with Miss Stanford was of the slightest, laid a hot, nervous grasp upon her neighbour's arm and said—

"Oh, Miss Stanford, do you really think there is any danger."

"Not the very slightest," was Beatrice's reassuring reply.

"It would be most unfortunate if any outbreak took place now. Our doctor has just left Mayfield, and we have not yet chosen another. There are so many here, and it is impossible almost to choose amongst them and to inquire among one's friends only increases one's bewilderment; every lady whose advice I ask says her medical man is *the* medical man in Mayfield. Who is yours, Miss Stanford, if I may ask?

"I havn't one," said Beatrice, in her frank way. "I am never ill. I have not thought of such a thing."

"But suppose you were taken suddenly ill, for whom should you send?"

"Really I don't know," knitting her brows a little impatiently, for she thought the conversation was rapidly becoming ridiculous. "I never was taken suddenly ill in my life," she concluded.

"Excuse my saying so," went on the other, and Beatrice's annoyance at the whole affair was increased when she found the group around her increasing, and smiles, more or less repressed beginning to appear in every face. She did not know that this lady had a kind of innocent mania on the subject of doctors, and in this way provided infinite amusement to her friends and acquaintances. "I feel as if I must speak," she went on, unmindful of the scorn on Miss Stanford's fine features, and the almost unconscious way in which she slightly withdrew from her. "Believe me you should lose no time in choosing your medical attendant and telling him your whole family history; after that one feels so safe and calm."

"I feel quite calm," said Beatrice, who had drawn herself up with some dignity, while this officious advice was being tendered.

"I shall always regret Dr. Furnival," went on the other. "He was a most reliable man, and had such a charming bedside manner."

In spite of her annoyance at being thus made into a butt Beatrice's sense of the ludicrous overcame her dignity at the last sentence; her eyes shone, and she had to bite her lip to restrain a smile, especially as every one else was now openly smiling. Her reply was therefore more good-natured than if it had come earlier it might have been.

"That must have been a great comfort to you," she said, "but, in justice to myself, I must say I hope I may never experience his charm." And disregarding the other's plaintive moan of "You never can, he's gone away," she went on, rising as she spoke to put an end to the interview, and thinking how Mrs. Ledward would have been amused by it.

"Pray do not feel anxious about me, I assure you there is no need. I am never ill; as far as my health is concerned I have not seen a doctor since I was a child."

She spoke in her usual clear penetrating tones, and as she did so some attraction drew her eyes upward, and she saw standing on the out-skirts of the group, which was enjoying the little scene, a man who looked at her with something of recognition in his eyes, and a smile, half amused, half cynical, upon his lips.

People were already turning away and no one was looking at her very attentively, or they might have been surprised to see how the colour rushed suddenly into her face and then died out again, leaving her very pale and with a strange startled look in her eyes. She stood in this way for a few seconds, and then, the man at whom she was looking having moved away, still with a slight smile curling his lips upwards, she, too, lowered her eyes and turned away in another direction.

That he had recognized her there could be no doubt; the expression on his face made that sufficiently clear, and she felt at once that the afternoon was spoiled for her. She sought comfort in the thought that she should never see him again; but she could not feel sure, he had already re-appeared where she had had no expectation of seeing him, and he remembered her as well as she remembered him, and he now had or believed himself to have knowledge, the thought of which caused Beatrice to feel a degree of shame and confusion which was unbearable. She could not forget his smiling cynical face, and although she knew he had gone away under a false impression, and she was accustomed in her superbly careless way to say that so long as she knew the truth about anything she cared nothing at all for what other people thought of it, there was something about this man which had impressed her in spite of herself, and she was angrily conscious that his opinion had weight with her. She walked about a little, dreading to see that face again, and, although she did not, pleasure was gone for her, and she felt only eager to get away. Out of consideration for Mrs. Ledward she tried to conquer the feeling, but in vain; and presently she beckoned to her side a youth who was standing near, and bade him go in search of Mrs. Ledward, and bring her to where Beatrice was sitting.

The gardens were large and crowded, and Laura Ledward was probably in the most remote corner of them, surrounded by her own particular coterie of admirers. A quarter of an hour passed before the youth returned accompanied by Mrs. Ledward, who talked gaily to him and filled his heart with joy. During that time Beatrice's proud, resolute face had assumed many expressions, but pleasure had not been amongst them, and she was looking almost stern as Laura approached.

"What is the matter, Beatrice?" asked Mrs. Ledward. "You are not thinking of going, are you?"

The stern look vanished. The beautiful face was only rather weary as Beatrice answered:

"Yes, I am, if you are ready. I am tired to death of it all. Such a noise and chatter."

Laura's face fell. She was enjoying herself thoroughly, and would have liked to stay to the end; but, in spite of Beatrice's scrupulously-polite form of "If you are ready," she knew what was expected of her, and said:

"Oh, yes, I am ready; but the carriage will not be here so soon."

"That does not matter," said Beatrice; "we can take a cab."

"What made you get tired so soon?" asked Laura, when they were seated at dinner an hour or two later. "I caught sight of you once and you seemed to be enjoying it."

"I did enjoy it at first, but I got bored. I have seen all those people so often, and besides that, I had seen a ghost."

"A ghost!" cried Laura, "in broad daylight, in a crowd at Mrs. Lennox's garden party! What was it like? Was it thin or fat? What colour was it? Could you see through it? Did it wear filmy garments, half hiding, half revealing its spectral shape, or was it clothed in ropes and rattling chains?"

Oh, no," said Beatrice quite gravely. "It was a perfectly nineteenth century ghost, clad in ordinary morning dress."

"Dear me!" said Laura, knitting her brows, "I am quite puzzled." Then suddenly her face cleared, and she went on eagerly—

"Did it take the form of a tall dark man, not handsome, but interesting-looking, with a good carriage and a keen, rather sarcastic face, and with a kind of upward twist of his lips when he smiled, which made him look not unlike one's idea of Mephistopheles?"

Beatrice looked at her in surprise, which was mingled with suspicion and a kind of fear. She was half inclined to deny the identity of her "ghost"; but her curiosity and the interest against which she fought all the harder as she felt herself inclined to yield to it, were too strong, and she said,

"Your description is highly exaggerated, but undoubtedly you have seen my ghost, too; what do you know about him?"

"Listen," said Laura impressively, "and I will tell you all. It was just before you sent young Davis after me. I had been talking to Sidney West and he had just gone to get me an ice, and I was sitting rather hidden behind my parasol, because the sun was shining straight into my eyes, when, suddenly, two men approached me. One was the individual whom I now know as your phantom, the other was a stranger to me, but he evidently knew you."

"That is very likely," said Beatrice, a little sharply, "To quote one of my favourite sayings, 'More people know Tom Fool than Tom Fool knows.'"

"Really, Beatrice," remonstrated Laura, "I wish you would not speak of yourself in that way, and do let me get on with my story. These men—if I had had any idea how interesting they would prove, I would have paid more attention to them, as it was, I only gave them the merest glance—these men, then came to a halt close to me, and I heard the phantom say:

"'But the lady in the yellow brown dress and the large picturesque hat, who is she?'

"'That is Miss Stanford," said the unknown, 'She has only lately come to live here. She is very beautiful, isn't she?'

"'She has a very beautiful face,' answered the phantom. I thought he spoke rather sarcastically, and I wondered why. You will excuse my entering into all these details, but I know your love of truth, and strive to satisfy it.

Beatrice said nothing, but made a little impatient gesture, and Laura hurried on.

"'Besides being beautiful,' went on the unknown, who in my opinion was rather coarse, "She is a double dyed heiress."

"'A *what*?' asked the phantom.

"'Her parents were both rich and she was an only child, and came in for all the money. She can't count her wealth, she owns ——'

"'My dear fellow,' broke in the phantom, and he spoke quite contemptuously, 'I don't want to know about Miss Stanford's belongings. I only asked who she was, because she is, as you say, very handsome, and I feel a little curious about her.'

"'Well,' said the unknown, 'I don't know her myself, but I know people who do; and I can get you an introduction if you like.'

"'I thank you,' said the phantom drily, 'but I don't care for introductions by favour, and I have no doubt I can continue to exist without basking in the tropic glow of Miss Stanford's smiles."'

"That last is your own touch, Laura," said Beatrice coldly.

"Well, perhaps it is," admitted Laura a little unwillingly, "I don't remember his own exact words, but they were to that effect. But who is he, Beatrice? what is his name and where did you meet him? Tell me all about it; how mean of you to keep it to yourself.'

"There is nothing to tell," said Beatrice with an effort. "I don't know his name, and I never have met him, only seen him once before. He reminded me of something very disagreeable, and I do not want ever to see him again.

"He seemed equally anxious to avoid you," said Laura cheerfully, "which is unusual. There is some mystery underneath it all I am sure."

"Laura, you are quite childish sometimes," said Beatrice impatiently, as she rose from the table and went back into the drawing room, whither Mrs. Ledward followed her baffled and curious.

Later in the evening Laura, feeling a little cross, for her party had been curtailed, and she felt instinctively that she was being held at arm's length in a matter which could not fail to prove interesting if she could only gain the slightest information about it, took refuge at the piano. In this way she earned Beatrice's gratitude. Miss Stanford felt just in the mood to sit and do nothing, yet after the conversation which had just taken place, she would not have yielded to this weakness under the very fire of Laura's keen and curious eyes.

She repeated to herself the conversation Laura ha reported, and her cheeks burned at the recollection. Bot men, whoever they might be, seemed to have spoken of her with a total absence of respect and delicacy; one had drawn attention to what she called her "money value," the other had emphasised her beauty of feature, as though money and physical charm were the highest attributes of a woman. He had acknowledged to feeling "curious" (odious word) about her, and had finally disclaimed all wish to make her acquaintance. She was in a mood to make the worst and the most of things. She did not spare herself one detail of the scene, or omit one qualifying word of Laura's as to tone, manner, and expression. When at last she went to her bedroom, she did not know whether she wished most never to see this man again or to make his acquaintance, and prove conclusively that he was wrong in his estimate of her.

(*To be continued.*)

